# Effects of Prosthetic Socket Design on Residual Femur Motion Using Dynamic Stereo X-Ray - A Preliminary Analysis

**DOI:** 10.3389/fbioe.2021.697651

**Published:** 2021-08-10

**Authors:** Jason T. Maikos, John M. Chomack, J. Peter Loan, Kathryn M. Bradley, Susan E. D’Andrea

**Affiliations:** ^1^ VISN 2 Biomechanics Research for the Advancement of Veteran Outcomes Laboratory, Veterans Affairs New York Harbor Healthcare System, New York, NY, United States; ^2^ C-Motion Inc., Germantown, MD, United States; ^3^ Virtual Reality and Motion Analysis Rehabilitation Laboratory, Providence VA Medical Center, Providence, RI, United States

**Keywords:** transfemoral amputation, biplanar videoradiography, socket kinematics, prosthetics, residual limb, dynamic stereo x-ray

## Abstract

Individuals with transfemoral amputation experience relative motion between their residual limb and prosthetic socket, which can cause inefficient dynamic load transmission and secondary comorbidities that limit mobility. Accurately measuring the relative position and orientation of the residual limb relative to the prosthetic socket during dynamic activities can provide great insight into the complex mechanics of the socket/limb interface. Five participants with transfemoral amputation were recruited for this study. All participants had a well-fitting, ischial containment socket and were also fit with a compression/release stabilization socket. Participants underwent an 8-wk, randomized crossover trial to compare differences between socket types. Dynamic stereo x-ray was used to quantify three-dimensional residual bone kinematics relative to the prosthetic socket during treadmill walking at self-selected speed. Comfort, satisfaction, and utility were also assessed. There were no significant differences in relative femur kinematics between socket types in the three rotational degrees of freedom, as well as anterior-posterior and medial-lateral translation (*p* > 0.05). The ischial containment socket demonstrated significantly less proximal-distal translation (pistoning) of the femur compared to the compression/release stabilization socket during the gait cycle (*p* < 0.05), suggesting that the compression/release stabilization socket provided less control of the residual femur during distal translation. No significant differences in comfort and utility were found between socket types (*p* > 0.05). The quantitative, dynamic analytical tools used in the study were sensitive to distinguish differences in three-dimensional residual femur motion between two socket types, which can serve as a platform for future comparative effectiveness studies of socket technology.

## Introduction

Following lower-limb amputation, the secure mechanical linkage of a prosthesis to the residual limb is essential to optimize function and comfort (Söderberg et al., 2003; Papaioannou et al., 2010) and is a key determinant for increased stability for successful ambulation (Legro et al., 1999; Kahle and Highsmith, 2014). For good coupling of the prosthetic socket to the residual limb, the interface between the bone and the prosthetic socket should have high stiffness during ambulatory activities to enable efficient dynamic load transmission from the distal prosthetic components to the residual limb (Söderberg et al., 2003). Different prosthetic socket designs and suspension methods offer distinct features to attain efficient control of the underlying tissue and bones (Eshraghi et al., 2014; Gholizadeh et al., 2014; Safari and Meier, 2015). However, relative motion between a socket and the residual limb, such as distal translation, or pistoning, is a common problem for individuals with lower extremity amputation (LEA) and can lead to higher incidences of secondary consequences, such as residual limb pain, skin breakdown, gait deviations, and reduced comfort (Eshraghi et al., 2012). Furthermore, poorly fitting sockets can increase risk of falls (Sawers and Hafner, 2021) and prosthetic abandonment (Sprunger et al., 2012), which can lead to lower health-related quality of life (Sinha et al., 2011).

Despite considerable improvements in prosthetic devices in recent years (Herr and Grabowski, 2012), socket technology has not kept pace with the developments in the field. Efforts have been made to digitize and automate socket design (Goh et al., 2005; Sengeh and Herr, 2013; Steer et al., 2020), though clinical practice has been slow to adopt these technologies and continues to rely on unscientific methods that lack repeatability (Sengeh and Herr, 2013). Novel socket designs offer the potential to improve the mechanical linkage between the residual limb and prosthetic socket. For example, compression/release stabilization (CRS) sockets incorporate alternating areas of compression and release through longitudinal struts and open windows to receive the displaced tissue (Alley et al., 2011). However, there is limited published research regarding the efficacy of CRS sockets and other novel socket designs in individuals with transfemoral amputation (TFA). Furthermore, the lack of time-efficient methods and analytical techniques to accurately quantify the three-dimensional (3D) residual limb-socket kinematics limits researchers’ ability to evaluate the complex biomechanical interactions between the residual limb/bone and socket. Ultimately, this limits the ability of clinicians to deliver evidence-based care to enhance socket fit.

To date, biomechanical assessments of the relative motion between the residual limb and prosthetic sockets have been suboptimal, using non-dynamic testing protocols (Bocobo et al., 1998; Kahle et al., 2020), static measurements (Commean et al., 1998; Gholizadeh et al., 2012), or unvalidated surface-marker-based motion capture systems (Eshraghi et al., 2012). Highly accurate, dynamic assessments of 3D, *in vivo* residual limb-socket kinematics are only possible using biplane videoradiography, also known as dynamic stereo x-ray (DSX), which can achieve sub-millimeter bone pose estimation accuracy for many functional movements (Miranda et al., 2011). Importantly, identification of a proper fitting socket is partially predicated on the analytical and experimental tools that aid in modeling bone position and orientation within a prosthetic socket. Since the movement of the residual limb within the socket is 3D in nature, it is important to utilize quantification techniques suitable for 3D measurement of dynamic movements to help quantify these *in vivo* movements. One study measured the residual limb-tissue-socket interface directly using DSX for individuals with transtibial amputation (Papaioannou et al., 2010), but the methods relied on time-intensive, subjective input, which can affect accuracy. Results of this investigation were also questioned for inaccuracies in maximum vertical slippage, possibly invalidating some of the techniques utilized (Eshraghi et al., 2011). Recently, Gale et al. utilized biplanar fluoroscopy for 3D markerless tracking of the residual femur during late swing and early stance to calculate the six degree-of-freedom kinematics of the residual femur relative to the socket. This study evaluated femur motion between a static pose and a separate recorded portion of the gait cycle during dynamic movement (Gale et al., 2020), but did not include terminal stance.

There remains a fundamental need to fill the gap of accurate, biomechanical evaluations of residual limb-socket kinematics for socket technology that can then be translated into evidence-based clinical practice. The purpose of this investigation was to determine the dynamic, *in vivo* kinematics between the residual limb and socket for individuals with TFA using two socket types: a CRS socket and a traditional, ischial containment (IC) socket. By providing a highly accurate, *in vivo* assessment of residual limb-socket motion in two different socket types, this vital foundational information can ultimately help enhance socket fit, while improving quality of life for individuals with TFA.

## Methods

### Participants

All study procedures were approved by the Institutional Review Boards of the Veterans Affairs New York Harbor Healthcare System (VANYHHS) and the Providence Veterans Affairs Medical Center (PVAMC). Five individuals with TFA were recruited for this study. Participants were at least 18 yr old and experienced prosthetic users (>6 h/day) without significant comorbid conditions or musculoskeletal limitations. All participants consented to participate prior to any study activities.

### Prosthetic Socket Fabrication, Fitting, and Evaluation

**FIGURE 1:**
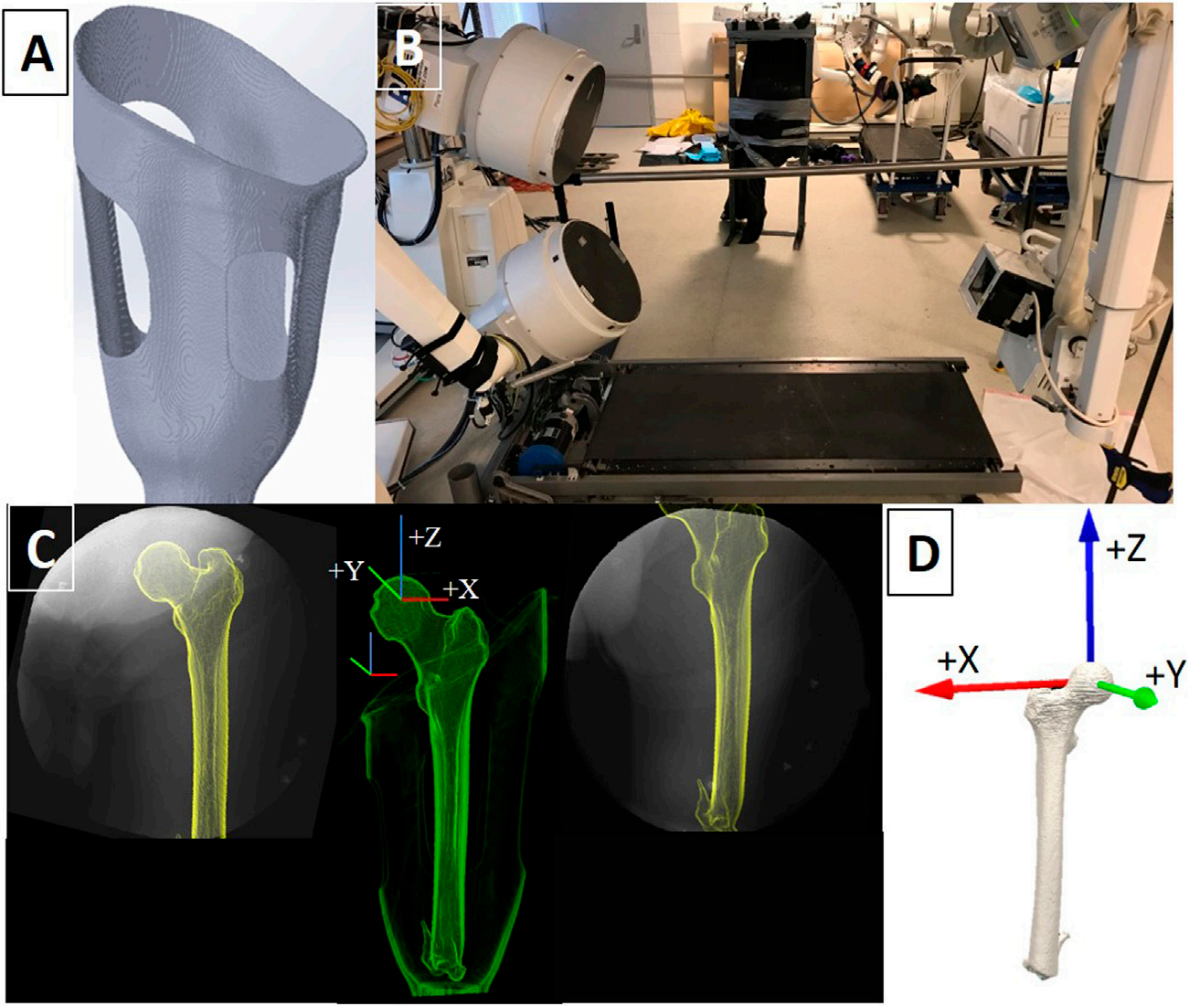
**(A)** Example of a digitally rendered transfemoral CRS socket with longitudinal depressions (struts) and open windows to accommodate the displaced tissue. **(B)** The DSX system was set up to record dynamic x-ray sequences in a 60° anteroposterior orientation as participants walked on the treadmill. **(C)** X-ray field of view images of the residual limb-socket complex from both x-ray views and the corresponding fusion of x-ray and CT imaging into a 3D entity. Femur and socket coordinate systems are displayed. **(D)** The anatomical coordinate system assigned to the residual femur was located at the center of the femoral head.

All participants had a well-fitting IC socket (i.e. traditional encapsulated socket) as determined by a board-certified prosthetist through standardized prosthetic guidelines. The IC sockets encapsulated the medial ischium and ischial ramus, which has been postulated to create a coronal “bony lock” with the pelvis to minimize lateral shifting of the femur during movement. CRS socket fabrication and fittings were performed by the CRS-certified study prosthetist. CRS socket fabrication utilized a compression jig to apply four longitudinal depressions in the socket walls with open release areas to receive the displaced tissue. This design aims to stabilize the residual bone through radial forces along the shaft of the femur, though limited research has been performed to assess socket efficacy (Alley et al., 2011) (Figure 1A). The study prosthetist ensured that the longitudinal depressions maximized contact pressure without overly reducing blood perfusion to the underlying tissues. Fit and comfort were confirmed through several “check sockets” before a laminated, definitive CRS socket was fabricated, fit, and aligned for each participant. Bench and static alignment were performed following manufacturer guidelines. Additionally, socket flexion and alignment were preserved from the existing IC sockets. Typically, the mechanical axes of the knee joints were aligned 15 mm posterior to a vertical reference line that bifurcated the socket wall at the ischium, while the midline of the feet fell 30 mm anterior to the reference line (Kobayashi et al., 2013). Each prosthesis was then dynamically tuned through software unique to each microprocessor knee, as well as to each participant’s gait pattern and adjusted to maximize functional mobility and safety. Participants used the same suspension system for both socket conditions: four participants used suction suspension and one participant used pin-locking suspension.

### Subjective Socket Evaluation

Participants were randomly assigned to start with either the traditional IC or CRS socket for 4 weeks of home use and then the process was repeated for the second socket. After each 4-week period, participants completed the utility subsection of Prosthesis Evaluation Questionnaire (PEQ) (Legro et al., 1998), the enabling factors section of the Prosthetic Profile of the Amputee (PPA) (Gauthier-Gagnon and Grisé, 1994; Franchignoni et al., 2007), and the functional satisfaction subscale of Trinity Amputations and Prosthesis Experiences Scale-Revised (TAPES-R) (Gallagher and MacLachlan, 2004; Gallagher et al., 2010).

### Dynamic Stereo X-ray Data Collection and Processing

A randomized, experimental crossover design was performed to compare differences between the prosthetic socket types. Each socket was used at home for 4 weeks. At the end of the 8-week trial, each participant underwent DSX testing on both sockets to quantify the 3D residual bone kinematics from biplane radiographic images. All data were collected at the W.M. Keck Foundation X-Ray Reconstruction of Moving Morphology (XROMM) facility at Brown University. The XROMM DSX system, previously described in detail (Miranda et al., 2011), contains two X-ray sources, which were positioned with beam paths intersecting at 60° in a plane path parallel to the floor (Figure 1B). DSX data were collected with an exposure of 1000−1500 µs, 95−105 kVp, and 200 mA at a resolution of 1760 × 1760. These setting were derived from Miranda et al. (Miranda et al., 2011) and modified as a result of tissue density. Validation of the XROMM DSX system for tracking the distal femur in static and high-speed impact conditions by Miranda (Miranda et al., 2011, Miranda et al., 2013) demonstrated systemic errors of 0.1–0.25 mm in translation and 0.1–0.3° in rotation for marker-based tracking, and sub-millimeter translation and 0.14-degree rotation errors for 3D volumetric model-based tracking. Image de-distortion and 3D space calibration were performed using previously described methods (Brainerd et al., 2010).

For each socket condition, participants were positioned on a treadmill within the biplane system so that the prosthesis and bony landmarks would remain optimized within the 40.6 cm diameter field of view (FOV) of each of the Image Intensifiers (II) throughout the entire gait cycle (Figure 1C). Participants walked on a treadmill at the same self-selected speed for each socket condition. Four walking trials per socket type were collected. DSX data were collected simultaneously with a six-camera Qualisys (Gothenburg, Sweden) passive, optical motion capture (OMC) system. OMC was used to record the overall position and orientation of the lower limbs and pelvis that were out of the DSX FOV. DSX data were time-synchronized with OMC using an electrical trigger and collected at 120 Hz during each walking trial.

### Quantification of the 3D Position and Orientation of the Residual Femur

A computed tomography (CT) scan of the residual femur without prosthesis (in the supine position) and CT scans of each socket were acquired for each participant (GE Lightspeed 16 CT Scanner; General Electric, Milwaukee, WI, United States). CT images were acquired at a resolution of 0.22 × 0.22 × 0.625 mm^3^ (80 kVp, SMART mA). Calibration and processing of all x-ray and CT data were performed within DSX Suite (C-Motion, Germantown, MD, United States). The residual femur was segmented from the CT volume and used to construct a polygonal mesh representing the bone surface. All global and local coordinate systems for DSX, OMC, and reconstructed CT data used a right-handed coordinate system with +Y anterior, +Z superior, and +X perpendicular to YZ plane. The local coordinate system for the CT-based residual femur model was located at the center of the femoral head, matching the local coordinate system of the femur in the motion capture model and socket coordinate system (Figure 1D). The axial plane and +Z axis were oriented to be parallel to the femoral shaft, following the direction of positioning expressed during gait. The +Y direction follows the suggested direction of progression by the International Society of Biomechanics. The 3D poses (position and orientation) of the femur were calculated using a model-based tracking algorithm that matched digitally reconstructed radiographs (DRRs) to x-ray images in two non-coplanar views (Bey et al., 2006; Anderst et al., 2009).

DRRs were generated by positioning the segmented CT volume within a virtual x-ray system and projecting rays through it to create simulated x-ray images. The optimal pose of the bone maximized the similarity between DRR images and their corresponding x-ray images. The x-ray images were smoothed with a Gaussian filter, then enhanced with an edge-detection convolution. The DRR images were enhanced with an edge-detection convolution, then scaled to match the intensity range of the x-ray images. Before tracking each motion trial, the femur was positioned manually in select time frames, typically every 4th to 10th frame of the high-speed trials (120 Hz). These poses served as key frames for cubic splines that covered the entire trial. The placement of key frames was done heuristically so that the splines provided a good approximation of the femur poses for all frames. The splines were then evaluated by the tracking algorithm to determine an initial pose of the femur for each frame. The output was 4 × 4 transformation matrices representing the poses of the residual femur in the motion capture coordinate system, which were exported to Visual3D (C-Motion, Inc., Germantown, MD, United States). The poses of the socket were calculated for each time frame using surface markers placed on the rigid exterior. Excursions for relative femoral rotations and translations were defined as the difference between the maximum and minimum values for each variable within the gait cycle.

### Data Analysis

Within-subject differences between the relative residual bone movement for the traditional IC and CRS sockets were determined in six DOF for each participant. Wilcoxon signed-rank tests were performed to compare differences in femur translations and rotations (IBM SPSS, Armok, NY, United States). Non-parametric analysis was performed due to the small sample size and less reliance on assumptions. A 95% confidence interval was calculated using a t-statistic and the two sample means to generate an interval estimate of the difference between the two population means. A mixed effects model was utilized (R, Vienna, Austria) to quantify the associations between repeated outcome measures and socket type, as well as to control for potential confounding factors (gender, age, cause of amputation, time since amputation).

## Results

### Participant Demographics

All participants wore a microprocessor-controlled knee and four participants used energy storing and return (ESR) prosthetic feet, while one participant used an articulating ESR prosthetic foot. The average age was 48.8 ± 11.9 yr, the average weight was 77.8 ± 11.8 kg, and the average time since amputation was 16.3 ± 14.3 yr. Four of the amputations were due to trauma and one amputation was due to cancer (Table 1).

**TABLE 1:** Participant Demographics.

Participant	Sex	Amputation Side	Age (yrs)	Height (m)	Weight (kg)	Time Since Amputation (yrs)	Suspension Method	Prosthetic Knee	Prosthetic Foot	Cause of Amputation
1	F	L	61	1.5	54.4	39.0	Suction	Plié	Kinterra	Trauma
2	M	R	42	1.7	88.5	3.1	Suction	X3	Rush	Trauma
3	F	R	60	1.6	61.2	19.9	Suction	Genium	Celsus	Cancer
4	M	R	33	1.8	78.9	5.8	Suction	X3	Triton	Trauma
5	M	R	48	1.7	82.6	13.5	Pin	X3	Rush	Trauma
Average (SD)			48.8 (11.9)	1.7 (0.1)	77.8 (11.8)	16.3 (14.3)				

Note: Data are mean (SD). F, Female; M, Male; L, Left; R, Right.

### Residual Limb Rotations and Excursions

**FIGURE 2:**
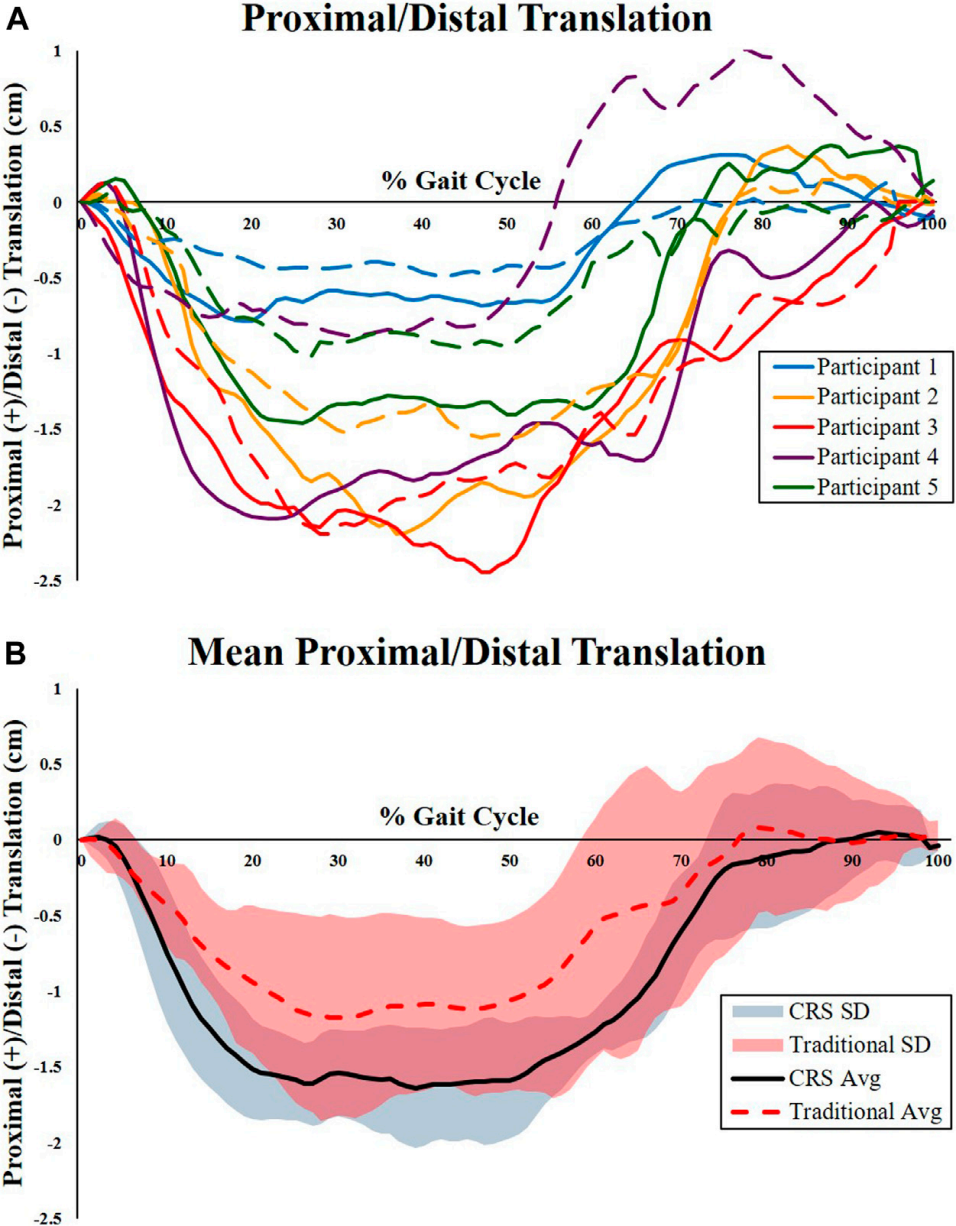
Proximal-distal kinematics of the residual femur relative to the prosthetic sockets. **(A)** Individual kinematic proximal-distal plots of the femur for each participant relative to the corresponding socket type. Dashed lines represent relative femur movement in the traditional IC socket. Solid lines represent relative femur movement in the CRS socket. Participant 5 (Green line) used pin locking suspension. All other participants used suction suspension. **(B)** Mean and standard deviation of the proximal-distal kinematics of the residual femur relative to the traditional IC socket (dashed line) and CRS socket (solid line). The traditional socket had significantly less proximal-distal translation compared to the CRS socket.

Total excursion of the residual limb rotations and translations, defined as the difference between the maximum and minimum values within the gait cycle, are shown in Table 2. There were no significant differences found between socket types for the three rotational degrees of freedom (anteroposterior rotation, medial-lateral adduction and abduction, and internal and external rotation). Furthermore, no significant differences were found between the anteroposterior and medial-lateral femur translations between sockets. Significant differences between proximal-distal translation (pistoning) were found between the traditional IC and CRS sockets. Figure 2 represents the proximal-distal plots of the residual femur for each participant relative to the corresponding socket type (Figure 2A) and the mean and standard deviation of each socket type (Figure 2B). The CRS socket demonstrated greater proximal-distal translation (2.0 ± 0.6 cm) compared to the traditional socket (1.6 ± 0.7 cm; *p* = 0.04)*.* Results from the mixed effects model did not show a strong effect from potential confounding factors (*p* > 0.50).

**TABLE 2:** Femoral Rotations and Translations by Socket Type and Survey Scores.

	CRS	Traditional	95% CI of difference in means	*p*-value
Flexion (+)/Extension (−) (degrees)	16.0 (9.7)	15.4 (6.4)	−11.4 to 12.6	0.89
Adduction (+)/Abduction (−) (degrees)	13.0 (5.6)	10.8 (5.3)	−5.9 to 10.1	0.08
Internal (+)/External (−) Rotation (degrees)	16.6 (7.1)	14.8 (9.9)	−10.8 to 14.4	0.35
Anterior (+)/Posterior (−) translation (cm)	2.1 (0.6)	2.4 (0.8)	−0.7 to 1.4	0.23
Lateral (+)/Medial (−) translation (cm)	3.7 (1.3)	4.6 (2.2)	−1.7 to 3.5	0.14
Proximal (+)/Distal (−) translation (cm)	2.0 (0.6)^a^	1.6 (0.7)	−0.4 to 1.4	0.04
**Subjective survey scores by socket type**				
Tapes-R functional satisfaction	10.8 (2.6)	8.2 (1.5)		0.63
PPA enabling factors	53.8 (3.0)	54.4 (2.3)		0.63
PEQ utility subscale	73.1 (23.1)	58.0 (11.6)		0.50

Note: Data are mean (SD). CRS, Compression/Release Stabilization; CI, Confidence Interval; PPA, Prosthetic Profile of the Amputee; PEQ, Prosthesis Evaluation Questionnaire.

a Significantly different than the Traditional Socket.

### Subjective Outcomes

There were no significant differences found between socket types for TAPES-R functional satisfaction subscale (*p* = 0.63) (Table 2). Similarly, differences between PEQ utility subscale scores for the CRS (73.1 ± 23.1) and traditional IC (58.0 ± 11.6) sockets did not reach statistical significance (*p =* 0.50). The PPA enabling factors subscale scores were similar for both the CRS (53.8 ± 3.0) and traditional IC (54.4 ± 2.3, *p* = 0.625) sockets.

## Discussion

The secure attachment of a prosthetic socket to the residual limb is critical for user satisfaction (Legro et al., 1999), quality of life (Pezzin et al., 2004), and reduction of secondary comorbidities (Gailey et al., 2008). This study demonstrated that the traditional IC socket design had significantly less proximal-distal translation compared to the CRS socket for individuals with TFA during treadmill walking at self-selected speed. Kinematics in three rotational DOF, as well as anterior-posterior and medial-lateral translation, were not significantly different between each socket type. Though this study indicated a significant difference in the proximal-distal translation of the residual femur between the socket conditions, it should be cautioned that investigations conducted with small samples sizes can be prone to a higher prevalence of Type II errors, as well as have limited generalizability of the results. To account for the small sample size, non-parametric analyses, which rely on less assumptions, were performed to compare differences in femur translations and rotations.

The multi-factorial nature of socket fit is a challenge that is complicated by the interaction between the residual limb, the liner, and the socket during dynamic activities. The quality of socket fit can be affected by individual residual limb characteristics, such as soft tissue and subcutaneous fat distribution (Webster et al., 2012), muscle re-assignment during amputation (Gottschalk, 2016), among many other factors. Improper fit or inappropriate prescription can cause increased relative movement of the residual limb within the socket and result in pain, negative limb tissue responses to external mechanical loads at the socket interface (Reynolds and Lord, 1992), and skin breakdown caused by tissue deformation and shear, all resulting in overall discomfort (Samitier et al., 2016). Research has shown that even subtle joint translations or rotations detected through advanced diagnostic imaging are critical to estimating key clinical measures such as tissue stress, joint impingement, or implant kinematics (Hoshino and Tashman, 2012; Farrokhi et al., 2015; Zhao et al., 2015; Lin et al., 2016), especially during dynamic movements. The same level of accuracy may be required for assessing the complex residual limb dynamics to optimize prosthetic socket design.

The increased proximal-distal translation of the residual femur in the CRS socket quantified in this investigation may be partially explained by design features of the socket. The CRS socket incorporates alternating areas of compression and release through longitudinal struts and open windows to receive the displaced tissue. The struts push into the underlying tissue creating radial forces, which have been postulated to help stabilize the bone (Alley et al., 2011). The manufacturer claims that these radial forces “pre-compress” the underlying tissues, thereby reducing the amount of tissue that needs to be compressed for a more efficient load transfer to the prosthetic socket. This was noted in a case series of participants with transhumeral amputation (THA), in which one participant showed considerably less rotation and translation of the humerus compared to the traditional socket during humeral abduction (Resnik et al., 2016). However, there are significant differences in the size and composition of underlying tissues of the residual humerus compared to residual thigh. Though not measured in this study, the radial forces and “pre-compression” of soft tissue induced by the CRS socket in this study may have had a negligible effect on stabilizing the proximal-distal translation of the residual femur during walking, which may be due in part to the larger amount and different composition of underlying tissue. Prosthetic sockets typically do not offer significant axial “pre-compression” of the distal tissues of the residual limb. Therefore, as axial load is applied during the stance phase of walking and rapid deceleration of the stance limb occurs, the distal tissues of the residual limb must first compress into the distal socket prior to the transfer of load to the prosthetic socket, resulting in distal translation of the femur. As the anterior musculature of the residual limb contracts during early to mid-stance to control vertical deceleration and to absorb the impact forces at heel strike, it is possible that the observed increased distal translation of the residual bone was caused by inadequate surface bearing of the struts and cutouts of the CRS socket to control the displacement of the residual tissue, allowing increased “distal settling” of the residual limb. To reduce “distal settling” and improve control of the residual limb for the CRS socket, it may be critical to increase the localized pressure along the longitudinal depressions of the socket, but this would likely cause, at minimum, an uncomfortable or intolerable socket environment, or worse, severely reduce blood perfusion to the soft tissue. A well-fitting encapsulated socket may provide better surface area at the liner/tissue-socket boundary, providing better anchoring of the residual tissue to the liner and socket, reducing “distal settling” of the tissue (Hachisuka et al., 1998; Kahle and Highsmith, 2013); however, this was not evaluated as part of this investigation. This increased anchoring could therefore reduce the amount of axial translation of the residual femur prior to load transmission to the proximal joints. Future clinical investigations or simulations should evaluate the amount of radial force and contact pressure needed to stabilize the residual femur to reduce distal compression, as well as determine if this contact pressure exceeds known threshold values of pain and skin breakdown (Meng et al., 2020).

Similar kinematics in the three rotational DOFs were observed between the two socket types. This suggests that the CRS socket did not provide additional rotational stabilization of the femur due to “pre-compression” of the underlying tissues of the residual limb, unlike the observed results in the THA case study. This may be due again to the larger diameter of the thigh musculature, which may require significant radial pressure to effectively “pre-compress” the soft tissue of the residual thigh. It is possible the amount of radial forces needed to effectively stabilize the femur may not be tolerable to the patient or may lead to tissue ischemia. The radial forces imposed by the CRS struts were not measured in this study. As such, future “pressure-dosing” studies should be conducted for CRS sockets to optimize femur stabilization.

Projected trends indicate that the overall number of lower limb amputations will increase dramatically, largely attributable to the aging population and the number of people living with dysvascular disease and diabetes (Ziegler-Graham et al., 2008). With this already large population expected to grow, considerable resources will be required for prosthetic services. By utilizing advanced analytical tools for a highly accurate, *in vivo* assessment of residual limb-socket motion, vital foundational information can be provided to aid in the evaluation of current technology, as well as the development of new methods and techniques to enhance prosthetic fit. The ability to accurately assess the inherent dynamic interaction between the residual limb and socket is necessary to develop effective, evidence-based prosthetic solutions to reduce secondary comorbidities and degenerative changes that result from complications of poor prosthetic load transmission.

## Limitations

This investigation included a small number of participants with a wide range of ages, prosthesis experience, time since amputation, and ankle-foot devices. Future research should standardize the prosthetic componentry to reduce variability. While prosthesis alignment was preserved between socket conditions to accommodate intra-subject comparisons in this study, future work should consider standardizing prosthesis alignment between participants. Furthermore, a larger sample size would increase the generalizability of results to a larger population of individuals with LEA, as well as reduce the possibility of type II errors. The small sample size may have also prevented the identification of significant relationships between femur motion and socket type. The femur was manually positioned at every 4th to 10th time frame, which may cause intra-tracker errors that could be improved with automated algorithms. Some potential confounding factors, including measurements of the residual limb (i.e. length, circumference, percentage of the sound limb), were not collected and should be explored in future work. Additionally, mechanical loading measurements of the residual limb were not collected as part of the study protocol, which could further the biomechanical understanding of the residual limb-socket interface. While several subjective surveys were included in the analysis, inclusion of the prosthetic socket fit comfort score (Hanspal et al., 2003) in future work could help explore potential correlations between pain and residual femur motion. Lastly, DSX has a limited FOV, which is unable to record the overall position and orientation of the lower limbs and pelvis that were out of view.

## Conclusion

Individuals with TFA often experience relative motion between their residual limb and prosthetic socket, causing inefficient dynamic load transmission through the body, compromised gait patterns, and secondary comorbidities that affect quality of life. Findings from this study indicated that the traditional IC socket design demonstrated significantly less proximal-distal translation of the residual femur compared to the CRS socket during treadmill walking at self-selected speed. Rotational kinematics of the residual femur in the sagittal, coronal, and transverse planes, as well as anterior-posterior and medial-lateral translation, were not significantly different between socket types. By utilizing highly accurate *in vivo* analytical tools, vital information can be measured to allow clinicians the ability to deliver the most effective evidence-based care for individuals with LEA.

## Data Availability

The raw data supporting the conclusions of this article will be made available by the authors, without undue reservation.
